# Autophagy and Akt in the protective effect of erythropoietin helix B surface peptide against hepatic ischaemia/reperfusion injury in mice

**DOI:** 10.1038/s41598-018-33028-3

**Published:** 2018-10-02

**Authors:** Rumeng Tan, Hongzhe Tian, Bo Yang, Bo Zhang, Chen Dai, Zhenyi Han, Meixi Wang, Yakun Li, Lai Wei, Dong Chen, Guangyao Wang, Huifang Yang, Fan He, Zhishui Chen

**Affiliations:** 1 Institute of Organ Transplantation, Tongji Hospital, Tongji Medical College, Huazhong University of Science and Technology, Wuhan, 430030 China; 2 Key Laboratory of Organ Transplantation, Ministry of Education, Wuhan, China; 3 NHC Key Laboratory of Organ Transplantation, Wuhan, China; 4 Key Laboratory of Organ Transplantation, Chinese Academy of Medical Sciences, Wuhan, China; 5 Department of Organ Transplantation, Changhai Hospital, Second Military Medical University, Shanghai, P. R. China; 6 Department of nephrology, Tongji Hospital, Tongji Medical College, Huazhong University of Science and Technology, Wuhan, 430030 China

## Abstract

Helix B surface peptide (HBSP) is an erythropoietin (EPO)-derived peptide that protects tissue from the risks of elevated blood pressure and thrombosis. This study focused on the protection of HBSP in hepatic ischaemia/reperfusion (I/R) by enhancing the level of autophagy. In detail, we randomly divided C57BL/6 mice into sham-operated, hepatic ischaemia/reperfusion (I/R), I/R + HBSP, I/R + HBSP + 3-methyladenine (autophagy inhibitor), I/R + HBSP + rapamycin (mTOR inhibitor), and I/R + HBSP + Ly294002 (Akt inhibitor) groups. We assessed alanine aminotransferase (ALT), aspartate aminotransferase (AST) and lactate dehydrogenase (LDH) levels in mouse sera, and performed haematoxylin/eosin (HE) staining, immunohistochemistry, electron microscopy, immunofluorescence microscopy, and western blotting on liver tissue to detect the degree of liver injury, liver apoptosis, autophagy, and the expression of microtubule associated protein 1 light chain 3 alpha (Map1lc3, or LC3), Beclin 1, phospho-mTOR, mTOR, phospho-Akt (P-Akt), and Akt. HBSP relieved hepatic I/R injury in a concentration-independent manner. The expression of LC3II, LC3I, and Beclin 1, and the formation of autophagosomes, in the I/R + HBSP group were higher than those in the I/R group. The protective effects of HBSP were abolished by 3-methyladenine and, to a lesser extent, Ly294002, but enhanced by rapamycin. Furthermore, *In vivo*, HBSP also protected against hypoxia injury induced by cobalt chloride (CoCl_2_) through improving the level of autophagy. Therefore, HBSP protected against hepatic I/R injury, mainly via regulating autophagy by targeting mTOR.

## Introduction

A large number of literatures have confirmed that Erythropoietin (EPO) has tissue protection and erythropoiesis. The erythropoietic effect of EPO has the risk of causing hypertension and thrombosis, which limits its application^1,2^. Erythropoietin (EPO) helix B surface peptide (HBSP) is a peptide derived from the 11-amino acid sequence of the waterborne surface of helix B in the tertiary structure of the EPO molecule^3^, as a derivative of EPO, retains the tissue protective effect of EPO and removes its role in erythropoiesis. As HBSP does not interact with Epor_2_^4^, unlike EPO, it does not promote the production of red blood cell^5^. Thus, it is not associated with the side effects of thrombosis and hypertension. HBSP plays a broad role in protecting tissues from injury^6^, as observed in the mouse kidney ischaemia/reperfusion (I/R) model^7^, a rabbit coronary atherosclerotic model^8^, and rat dilated cardiomyopathy^9^. HBSP mediates tissue protection by inhibiting inflammation and apoptosis during organ I/R injury^5,7^. It has several advantages over EPO as a tissue damage protection agent. However, its effects and potential mechanism in the liver have not been reported.

Hepatic I/R injury is a common cause of liver injury, especially during liver transplantation. The pathophysiology of liver I/R injury includes hypoxia-induced cell damage and inflammatory pathway activation caused by delayed disorders and injuries^10–15^. Recent studies have shown that autophagy is involved in major areas of liver disease. Since autophagy was first recognized due to its role during starvation, the study of autophagy in liver disease has focused on hepatic I/R injury^13^, during which it renders cells more effective in response to nutritional deficits and hypoxia. Autophagy is the main pathway for intracellular protein degradation. Autophagosomes fuse to lysosomes, then degrade intracellular longevity proteins and organelles to maintain nutrient stability under starvation or stress conditions, and to remove harmful cellular components^16–19^. Recently, a large number of studies have confirmed the protective role of autophagy in the kidney, lower limbs, myocardium, nerve tissue of multiple organs, and tissue affected by I/R injury^20–23^, but its role in the liver is still not clear^24^. Despite on-going research, it is also unclear how HBSP regulates autophagy to protect the liver from I/R injury. In this study, we uncovered the mechanism of HBSP protecting hepatic I/R injury.

## Result

### HBSP reduces liver I/R injury independent of concentration

**Figure 1:**
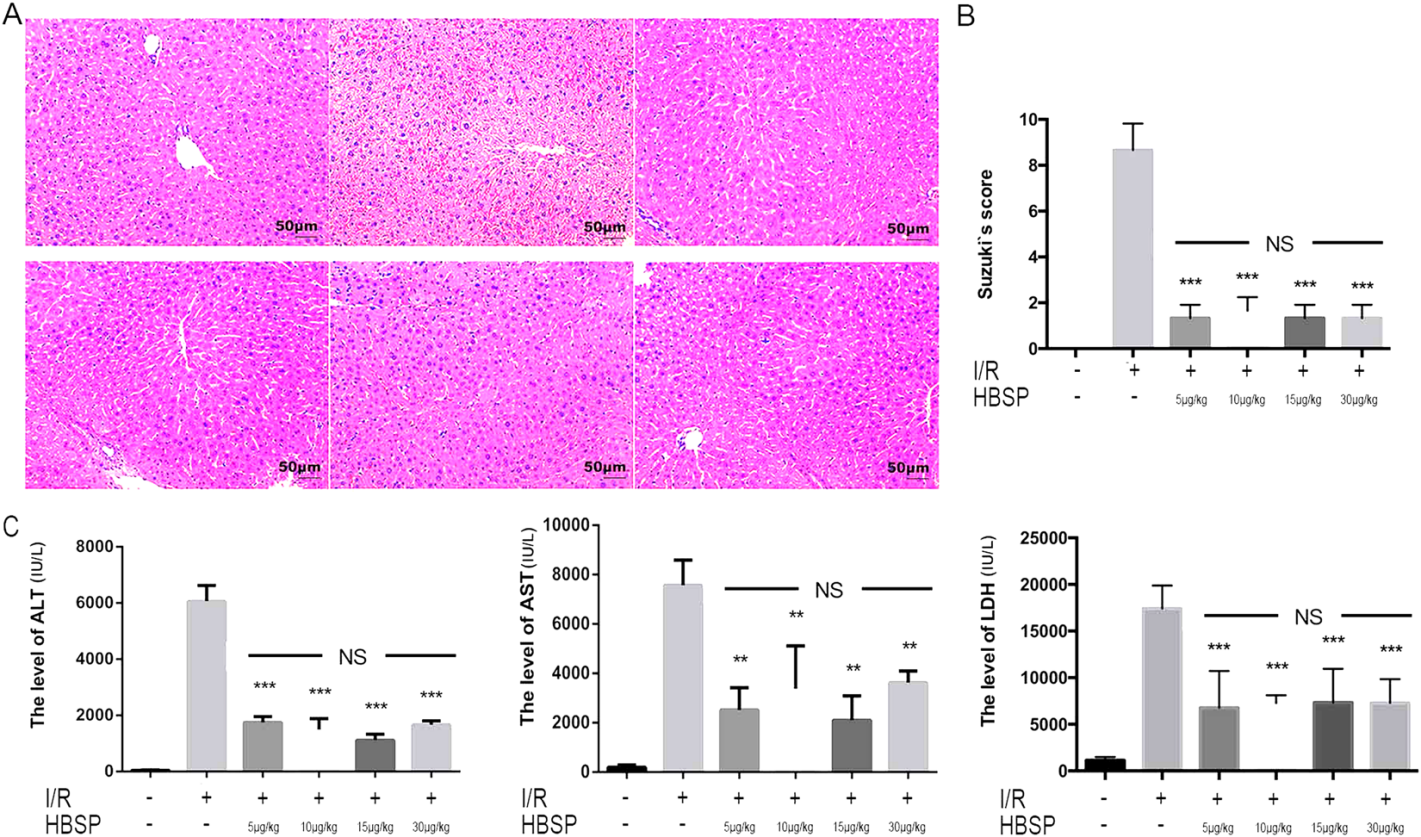
HBSP can significantly reduce I/R-induced liver injury in a concentration-independent manner. (**A**) Representative photographs (200×) of haematoxylin–eosin-stained liver sections from sham-operated, I/R, and I/R + HBSP mice. HBSP was injected into mice at the indicated concentrations. Scar bar, 50 μm. (**B**) Histopathological scoring of hepatic injury was performed. (**C**) Serum ALT/AST levels in sham, I/R, and HBSP-injected mice. *Significant difference from I/R group, **p < 0.01, ***p < 0.001; NS: Not statistically significant

Firstly, we observed the pathological changes in liver tissue. We found that the sham operation group showed normal liver tissue morphology, whereas the liver tissue in the I/R group had obvious inflammatory cell infiltration, haemorrhagic-induced changes, and a loss of tissue integrity. On the contrary, the degree of liver tissue damage was significantly reduced in the I/R + HBSP groups than that in I/R group, at all HBSP concentrations, and the inflammatory cell infiltration was decreased compared to in the I/R alone group (Fig. 1A). In addition, the ALT, AST and LDH levels in the I/R group were significantly increased over those in the sham group, whereas the levels of ALT and AST in the HBSP group were significantly relieved compared to those in the I/R group (Fig. 1B). These results suggest that HBSP significantly reduces I/R-induced liver injury independent of HBSP concentration.

### HBSP enhances autophagy in response to hepatic I/R injury

**Figure 2:**
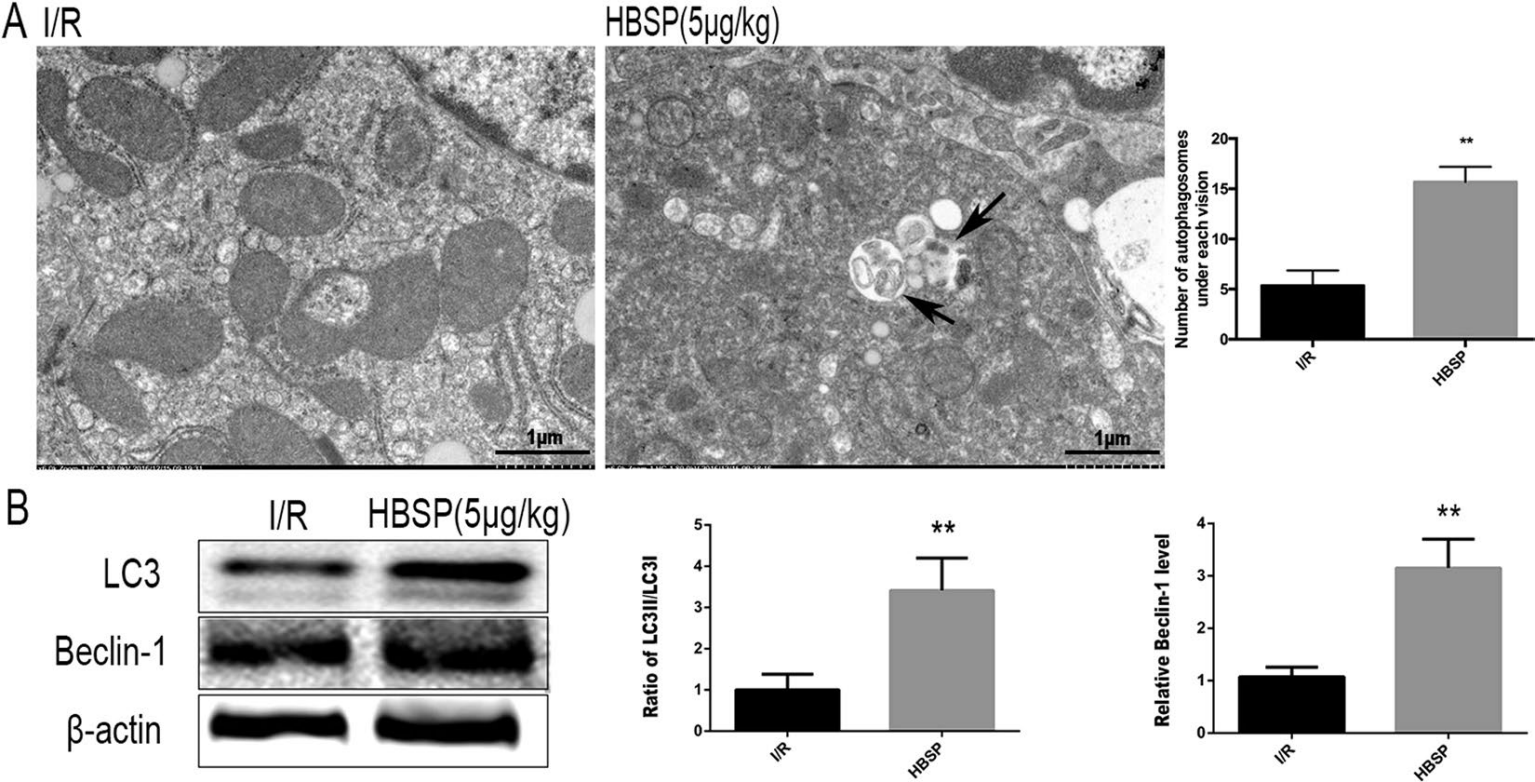
HBSP enhances autophagy during hepatic I/R injury. (**A**) Representative electron micrographs of autophagosomes. Scar bar, 1 μm. (**B**) Quantification of autophagosomes under EM. (**C**) Representative images of immunoblots for LC3I, LC3II, Beclin1, and β-actin (full-length blots are presented in the Supplementary Information file). **p < 0.01, compared to the I/R group.

Autophagy is accompanied by lipidation of microtubule associated protein 1 light chain 3 alpha (Map1lc3A, or LC3). Lipidation increases the expression of Beclin 1 and autophagosome formation^25^. Therefore, in this study, we assessed autophagy using the ratio of LC3II:LC3I, Beclin1 expression, and autophagosome formation. The ratio of LC3II:LC3I and the expression of Beclin1 were significantly higher in the HBSP group than those in the I/R group (Fig. 2B), and the number of autophagosome in the HBSP group was significantly higher than that in the I/R group (Fig. 2A).

### Inhibition of autophagy reverses the effects of HBSP in hepatic I/R injury

**Figure 3:**
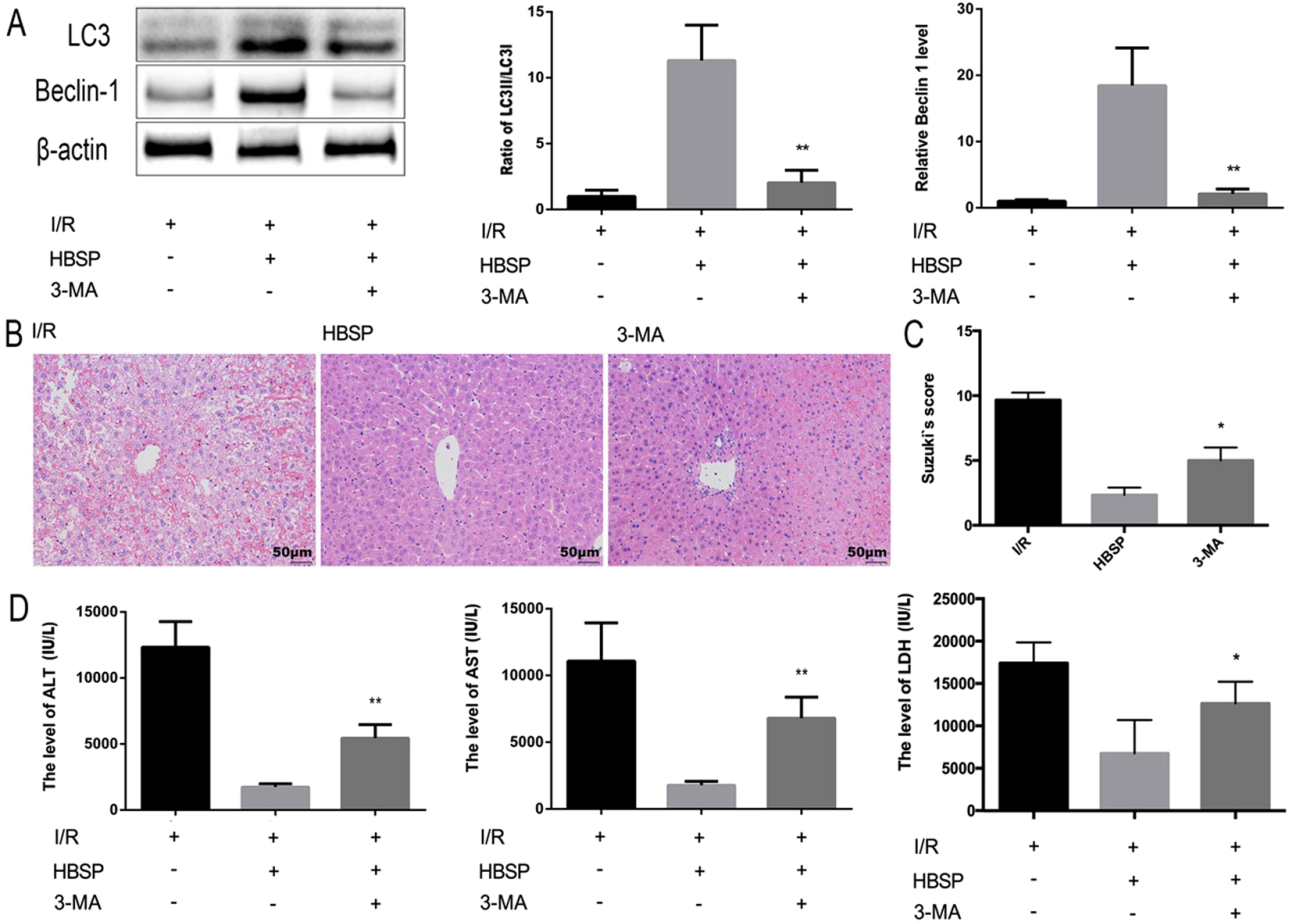
Inhibiting autophagy reverses the protective effects of HBSP in hepatic I/R injury. (**A**) Representative photographs of immunoblots against LC3I, LC3II, Beclin1, and β-actin (full-length blots are presented in the Supplementary Information file). (**B**) Representative photographs (200×) of haematoxylin–eosin-stained liver sections, Scar bar,50 μm. (**C**) Histopathological scoring of hepatic injury was performed. (**D**) Serum ALT and AST levels. *Significant difference from HBSP group, *p < 0.05, **p < 0.01.

The compound 3-methyladenine (3-MA) is commonly used as an autophagy inhibitor^26^. We found decreases in the LC3II:LC3I ratio and Beclin1 expression in the 3-MA group compared to the HBSP group (Fig. 3A). We also found that 3-MA increased the infiltration of inflammatory cells in the presence of HBSP and caused more severe liver damage than HBSP alone (Fig. 3B). In addition, the levels of ALT and AST in the 3-MA group were significantly higher than those in the HBSP group (Fig. 3C). Thus, the protective effect of HBSP against hepatic I/R injury is weakened by the inhibition of autophagy.

### Inhibition of mTOR enhances autophagy and strengthens HBSP-mediated protection against hepatic I/R injury

**Figure 4:**
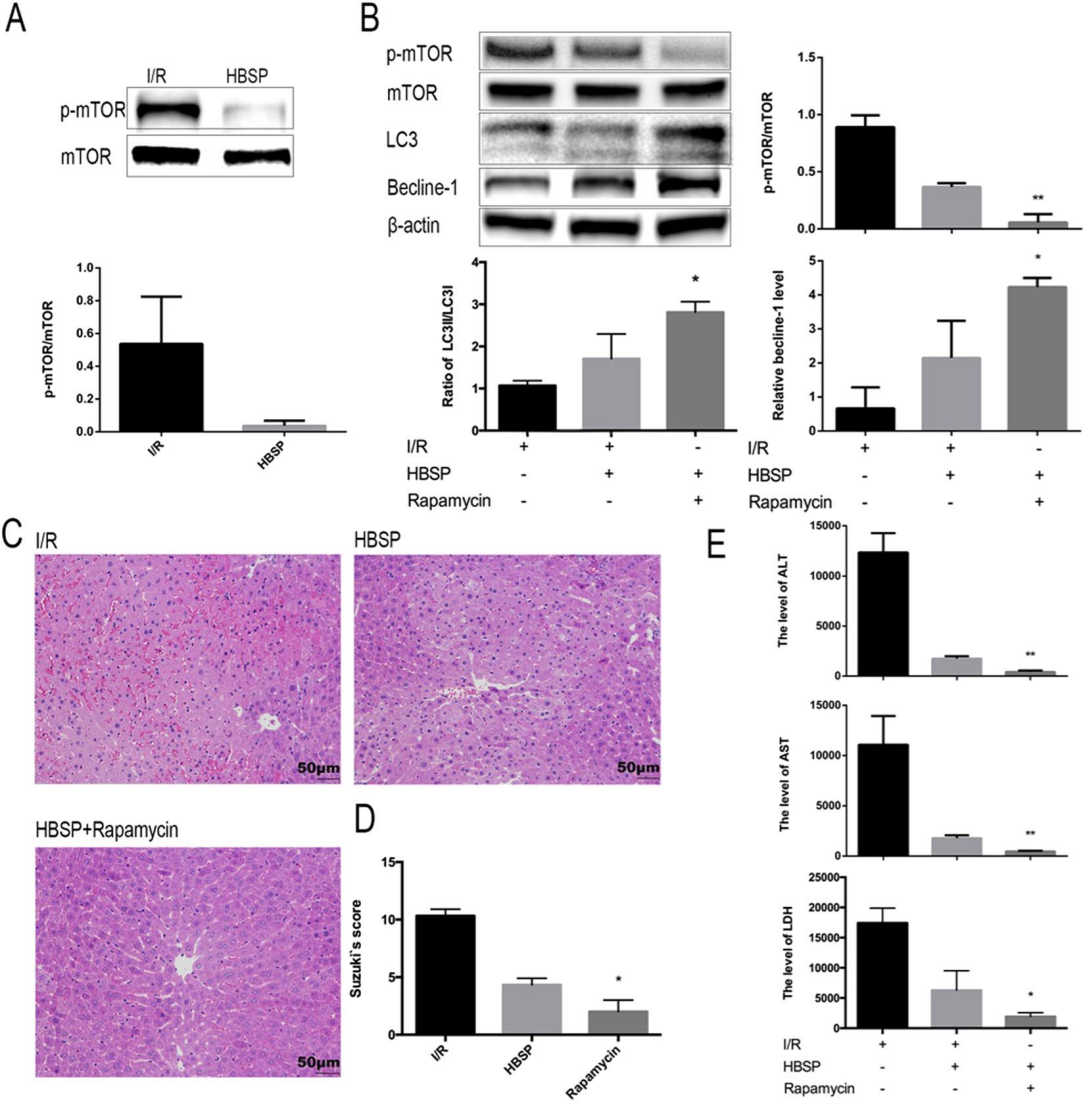
mTOR inhibition enhances autophagy and strengthens the protective effect of HBSP. (**A**) Representative photographs of immunoblots against phospho-mTOR and mTOR (full-length blots are presented in the Supplementary Information file) (**B**) Representative photographs of immunoblots against phospho-mTOR, mTOR, LC3, and β-actin. full-length blots are presented in the Supplementary Information file. (**C**) Representative photographs (200×) of haematoxylin–eosin-stained liver sections, Scar bar, 50 μm. (**D**) Histopathological scoring of hepatic injury was performed. (**E**) Serum ALT and AST levels. *Significant difference from HBSP group, *p < 0.05, **p < 0.01.

We found that mTOR phosphorylation levels in the HBSP group were significantly lower than those in the I/R group (Fig. 4A). Inhibition of mTOR phosphorylation with rapamycin^27,28^ reinforced autophagy (Fig. 4B), alleviated the infiltrated inflammatory cells (Fig. 4C), and intensified the ALT and AST levels (Fig. 4D) compared with the levels in the HBSP group. Together, these findings suggest that the protective effects of HBSP on the liver are bolstered by enhancing autophagy via inhibition of phospho-mTOR.

### HBSP partially reduces liver tissue apoptosis during hepatic I/R injury

**Figure 5:**
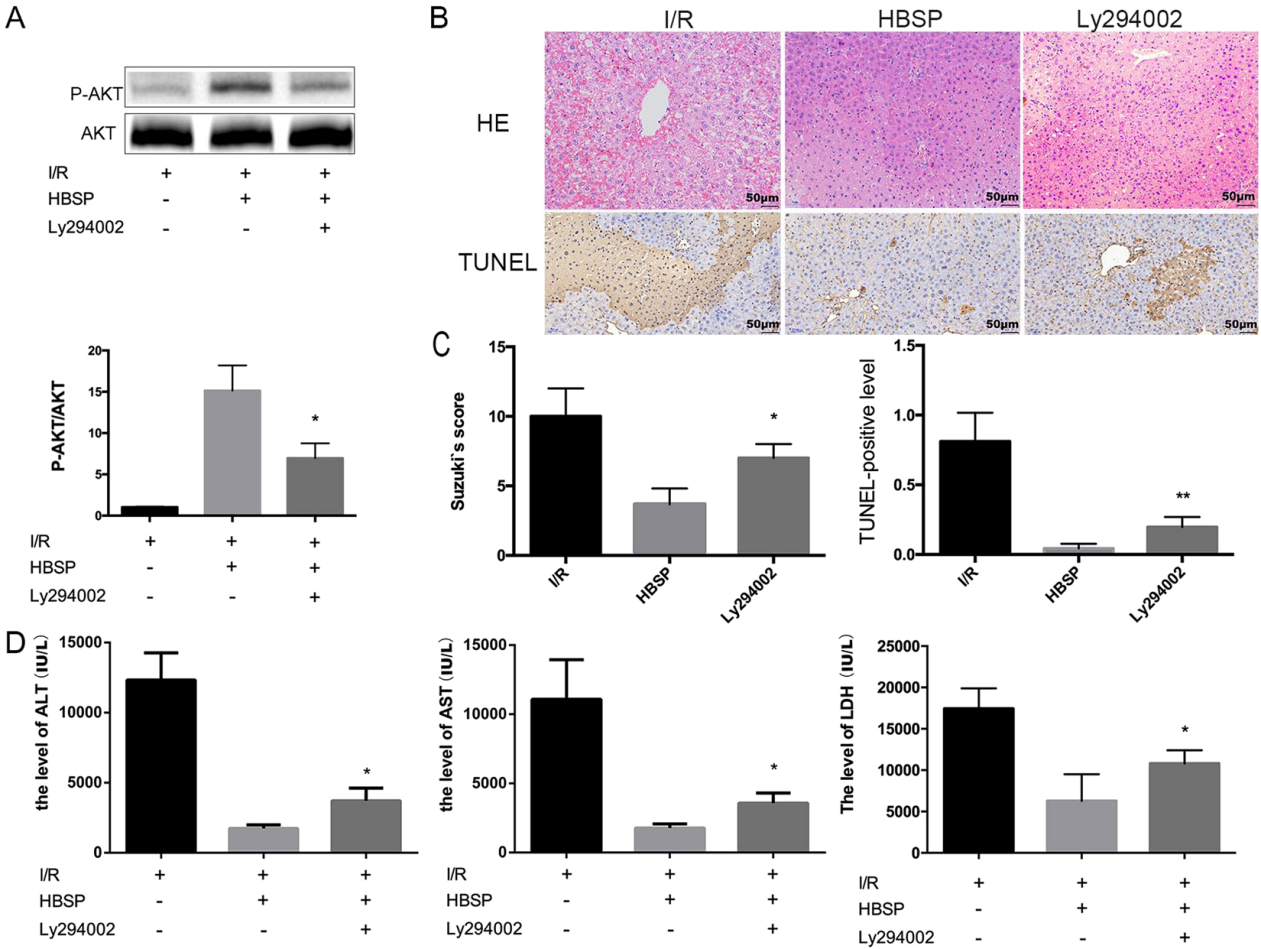
HBSP reduces liver tissue apoptosis during hepatic I/R injury. (**A**) Representative photographs of haematoxylin–eosin-stained section, and immunohistochemical staining for TUNEL in liver sections; Scar bar, 50 μm. (**B**) Serum ALT and AST levels. (**C**) Histopathological scoring of hepatic injury was performed and TUNEL-positive level was also quantificated. (**D**) Representative photographs of immunoblots against phospho-Akt and Akt (full-length blots are presented in the Supplementary Information file). *Significant difference from HBSP group; *p < 0.05, **p < 0.01.

Terminal deoxynucleotidyl transferase dUTP nick end labelling (TUNEL) was used to assess the degree of liver tissue apoptosis. Apoptosis of liver cells in the HBSP group occurred less frequently than those in the I/R group (Fig. 5A). The protective effect of HBSP on liver tissue was diminished by the Akt inhibitor Ly294002, and the degree of apoptosis was higher in the Ly294002 group than that in the HBSP group (Fig. 5B). However, comparing to 3-MA, which inhibited autophagy, the degree of liver function injury evaluated by ALT and AST in Ly294002 group was not that worse than that in 3-MA group. It may be inferred that autophagy play the primary role, and the activation of akt makes a partially contribution to the protection of HBSP in hepatic I/R injury.

### HBSP alleviates AML-12 cells hypoxia injury induced by CoCl_2_

**Figure 6:**
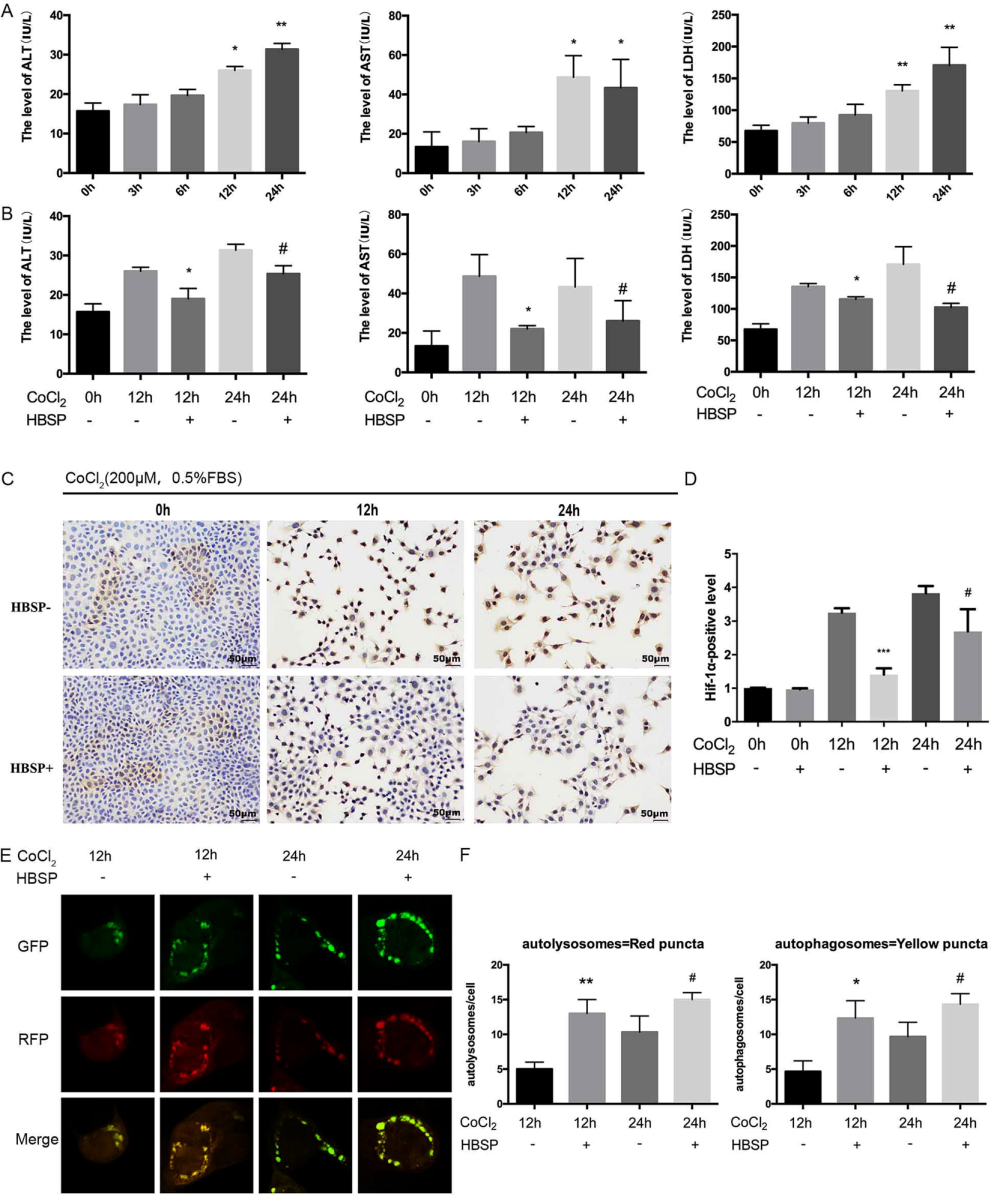
(**A**,**B**) AML-12 cells culture supernatant ALT, AST and LDH levels, *Significant difference from normal group (0 h). *p < 0.05, **p < 0.01. (**C**) Immunohistochemical staining for HIF-α in slides of AML-12 cells, Scar bar, 50 μm. (**D**) HIF-α positive level. (**E**) Representative image of GFP-LC3B puncta and mRFP-LC3B puncta in AML-12/mRFP-GFP-LC3 cells. (**F**) Quantification of LC3B positive autolysosomes or autophagosomes in AML-12/mRFP-GFP-LC3 cells. (**B**,**D**,**F**) *Significant difference from HBSP-/CoCl_2_ 12 h group, ^#^Significant difference from HBSP-/CoCl_2_ 24 h group; *p < 0.05, ^#^p < 0.05.

CoCl_2_ was often used as hypoxia mimic reagent. We found that the levels of ALT, AST, and LDH in the culture supernatant of AML-12 cells increased as the CoCl_2_ stimulation time increased (Fig. 6A) and the levels of ALT, AST, and LDH in the supernatants of cells treated with HBSP were lower than those without HBSP treatment (Fig. 6B). In addition, the expression of HIF-1α (a key transcription factor response to hypoxic stress)^29^ in cells treated with HBSP also decreased when compared with that in non-HBSP-treated cells (Fig. 6C,D). We also found that the level of autophagy in HBSP treated group was higher than that in non-HBSP treated group (Fig. 6E,F).

## Discussion

Early studies showed that autophagy played an important role in different I/R injury^30–33^ and increased autophagy was believed to have a beneficial effect on liver I/R injury^34–36^. Some features of hepatocytes were tightly dependent on autophagy^37^. Based on prior studies that autophagy played an unprecedented role in regulating cell function, as well as regulating liver lesions, by using genetic or chemical methods to block or increase the level of autophagy.

Recently, several studies have reported that HBSP displayed anti-inflammatory and tissue-protective activities in various I/R injuries^3^. However, the underlying mechanism remained largely unknown. HBSP mediates protective effects against myocardial I/R injury, chronic heart failure (CHF), and renal I/R injury^4,5,38^. But its role in hepatic I/R injury has not been reported in the literature. We found that HBSP protected against hepatic I/R injury via autophagy and Akt signalling.

Whether the activation of autophagy is the key mechanism of the protection conferred by HBSP in liver I/R injury remains unclear. Therefore, the aim of our study was to determine whether the autophagy pathway mediates the protective effects of HBSP treating in the liver. In our study, we found that HBSP could reduce hepatic I/R injury *in vivo* and the damage caused by hypoxia culture of hepatocytes *in vitro*. Simultaneously, HBSP resulted in increased autophagy in liver tissue *in vivo* and hepatocytes *in vitro*. The protective effects of HBSP in hepatic I/R injury were abolished with the inhibition of autophagy by 3-MA. These findings reveal that the hepatoprotective effect of HBSP against hepatic I/R injury depends, at least in part, on the activation of the autophagy. These findings indicate a critical role for autophagy in HBSP-mediated protection from hepatic I/R injury. Many molecules regulate and mediate autophagy, such as the ULK1 complex, mTORC1, AMPK, and TFEB^39^. There are literatures confirming that HBSP works through the mTOR pathway in disease, however its mechanism in hepatic ischaemia-reperfusion injury is not clear^40,41^. In order to identify the possible mechanism of HBSP in the regulation of hepatic I/R injury, we inhibited the activity of mTOR by Rapamycin. We found that rapamycin enhanced the protective effect and the level of autophagy in the model of HBSP protection hepatic I/R injuries. These results suggest that the mTOR pathway may be involved in the regulation of autophagy associated with HBSP-induced protection against hepatic I/R injury, and regulating mTOR-autophagy signaling pathway may be a way to reduce liver I/R injury.

In addition, we found that AKT was activatied by HBSP *in vivo* and HBSP can reduce liver tissue apoptosis. Furthermore, the combination of HBSP and Ly294002 treatment group contributed a lower hepatoprotective effect and relatively more severe apoptosis, indicating that Akt may be partially involved in the regulation of liver tissue apoptosis by HBSP during hepatic I/R injury.

However, there are still some limitations to our study that should be acknowledged. Firstly, the consequence of I/R injury include transient liver dysfunction and the systemic inflammatory response to syndrome, the indicators that reflected the systemic cytokines such as IL-6 or TNF levels in the sera from the blood samples of mice were not given. We only focused on the main outcome of I/R injury, which referred to the indicators widely used in clinical such as hepatic serum enzymes. Secondly, for some economic and technical limitations, the lack of autophagy gene knockout mice was an area of weakness for this study. So, in next research, we plan to screen out the key autophagy gene, then acquiring the right gene KO mice, and eventually could explain the molecular mechanism in depth of HBSP-protected the hepatic I/R injury.

In summary, our data demonstrated that HBSP mediated protective effects against hepatic I/R injury by regulating autophagy through the mTOR-autophagy pathway. This is the first time to clarify the key role of mTOR-autophagy signaling in regulating HBSP-mediated the protection of liver I/R injury. Another important implication of our results was that AKT was also an important factor in hepatic I/R injury. It is not clear whether the autophagy pathway and AKT pathway interact in this process.

Our study offers further evidence for the protective mechanism of HBSP in hepatic I/R injury, which has remained unclear despite numerous previous studies. This study also provides new targets for drugs to relieve hepatic I/R injury. However, the potential relationship between the different pathways involved requires further investigation.

## Methods

### Animals

The study was approved by the Institutional Animal Care and Use Committee at Tongji Medical College, Huazhong University of Science and Technology and all methods were carried out in accordance with the relevant guidelines and regulations of the Institutional Animal Care and Use Committee at Tongji Medical College, Huazhong University of Science and Technology. C57BL/6 mice were purchased from Beijing Vital River Laboratory Animal Technology, and maintained in the animal facility of the Institute of Transplantation, Tongji Hospital Affiliated to Tongji Medical College, Huazhong University of Science and Technology. At the time of experimentation, the mice were 8–10 weeks of age and weighed 22 g ± 2 g.

### Cell culture

For cell propagation, AML12 cell line (ATCC^®^CRL-2254^TM^) were grown in DMEM/F12 medium (Hyclone) containing 10% fetal bovine serum (Gibco), supplemented with 100 U/mL penicillin and 100 g/mL streptomycin (Gibco), and incubation under an atmosphere of 5% CO_2_. The cell hypoxia model was established by chemical regent cobalt chloride^42^ (CoCl_2_, Sigma, St. Louis, Missouri). The final concentration of CoCl_2_ was 200 μM. When CoCl_2_ was added to culture medium, the FBS final concentration was changed to 0.5%. Cells were harvested in the indicated time and then detecting the ALT/AST/LDH from the culture medium.

### Reagents

HBSP was synthesized at Shanghai Ketai Biotechnology. Rapamycin (9904) and Ly294002 (9901), and antibodies against LC3 (2775), Beclin1 (3495), β-actin (4967), phospho-mTOR (5536), mTOR (2983), p-Akt (4060), and Akt (4691) were purchased from Cell Signaling Technology. Antibodies against HIF-1α (ab82832) was purchaded from Abcam. Goat anti-rabbit IgG (7074) and goat anti-mouse IgG (7076) were purchased from Cell Signaling Technology. We purchased 3-MA (S2767) from Selleck Chemicals. Ad-mRFP-GFP-LC3 adenoviral was purchaded from DesignGene (Shanghai).

### Hepatic I/R model

In this study, we used a 70% hepatic I/R model. The blood vessels were occluded for 60 minutes to block the flow of blood to the left and middle lobes of the liver. The mice were sacrificed 6 hours after the blood vessels were opened. The sham operation group underwent only laparotomy without clamping of the blood vessels. Mice in the HBSP group were intraperitoneally injected with HBSP 12 hours before the operation, during the operation, and 3 hours after opening the blood vessel. The I/R group were injected intraperitoneally with an equal volume of saline at the same time points. Ly294002, 3-MA, and rapamycin were injected intraperitoneally into mice 0.5 hours before the administration of HBSP^11^.

### Serum sample assays

Serum ALT, AST and LDH levels were measured with a standard clinical automatic analyser in the clinical laboratory of Tongji Hospital affiliated to Tongji Medical College, Huazhong University of Science and Technology.

### Liver tissue pathology

We fixed fresh liver tissue in 10% paraformaldehyde, then dehydrated the tissue in a series of increasing alcohol concentrations followed by xylene. Then, the tissue was placed in paraffin wax, embedded, and stained with standard haematoxylin–eosin–safran and picrosirius. We captured images with a microscope (NIKON ECLIPSE CI). Histological evaluation was performed using a semi-quantitative scoring system^43^.

### Histological criteria for assessment of liver damage after I/R injury

The morphology results were assessed single blindly by a pathologist. In detail, the histological criteria for assessment of liver damage after I/R injury in this study was first introduced by Suzuki in 1993^44^. In brief, the assessment was considered in three aspects, including sinusoidal congestion, hepatocyte necrosis and ballooning degeneration. Each item was given the score from 0 to 4 depending on the degree of liver damage. When there were no congestion, vacuolization and necrosis, it was given a best score 0. Otherwise, if there were severe congestion accompanying with severe vacuolization and >60% necrosis area, the score was up to 12.

### Confocal microscopy

AML12 cell line was cultured on glass coverslip in 24 well plates and transfected with Ad-mRFP-GFP-LC3 adenoviral (MOI = 50) for 48 h followed by adding CoCl_2_ (final concentration 200 μM). The strength of the autophagic flow was determined by different fluorescence using a confocal scanning laser microscopy connected to the controlled computer. One of the fluorescence, mRFP was used to label and track LC3. Since the sensitivity of GFP fluorescent proteins to acidity, when autophagosomes were fused with lysosomes, GFP fluorescence was quenched and only red fluorescence was detected. So, the attenuation of GFP indicated the fusion stage of lysosomes and autophagosomes, which were formed into autolysosome occurred at the late autophagy stage. The yellow spots that appeared after we merged red-green double fluorescence after microscopic imaging were only autophagosomes. The red spots indicated autolysosome, and the intensity of autophagic flow could be clearly seen by counting the spots of different colors.

### Electron microscopy (EM)

The liver tissue was cut into pieces (1 mm × 1 mm × 2 mm), washed twice with saline, then fixed in glutaraldehyde for 2 hours. After rinsing, the tissue pieces were dehydrated in a series of increasing alcohol concentrations, then embedded, and cut into slices of 50–60 nm using the ultrathin slicer (Leica, LeicaUC7); photographs were taken with the transmission electron microscope (Hitachi, HT7700).

### TUNEL assay

Cell death in liver paraffin sections was detected by the TUNEL assay. The assay was performed according to the manufacturer’s instructions for the *In Situ* Cell Death Detection Kit (Roche, 11684817910).

### Western blot analysis

Briefly, 60 μg of extracted protein was subjected to separation by 12% SDS–PAGE and transferred to PVDF membranes (Millipore). Nonspecific binding sites were blocked with TBST with 5% bovine serum albumin for 1 hour at room temperature. The membranes were incubated with primary antibodies at 4 °C overnight, then washed and incubated with the secondary antibodies for 1 hour at room temperature, followed by washing and signal detection. The protein expression levels were quantified with Image-Pro Plus software (Media Cybernetics).

### Statistical analysis

All data are expressed as the mean ± SD. The data were analysed using a one-way analysis of variance followed by the least squares difference test (assuming equal variances) or Tamhane’s T2 test (without the assumption of equal variances). Comparisons between groups were performed with the two-tailed Student’s t test. A p-value < 0.05 was considered statistically significant.

## Electronic supplementary material

Full-length blots
