# Supplementary material for: Autophagy and Akt in the protective effect of erythropoietin helix B surface peptide against hepatic ischaemia/reperfusion injury in mice
Source: Sci Rep. 2018 Oct 2;8:14703. doi: 10.1038/s41598-018-33028-3 (PMC6168561; doi:10.1038/s41598-018-33028-3)
Supplement: Supplementary file 1 — Full-length blots [file 41598_2018_33028_MOESM1_ESM.docx]

**Autophagy and Akt in the protective effect of erythropoietin helix B surface peptide against hepatic ischaemia/reperfusion injury in mice**

Rumeng Tan, Hongzhe Tian, Bo Yang, Bo Zhang, Chen Dai, Zhenyi Han, Meixi Wang, Yakun Li, Lai Wei, Dong Chen, Guangyao Wang, Huifang Yang, Fan He, Zhishui Chen

We have appended full-length blots in the Supplementary Information file. In the figure legend, we have mentioned that full-length blots are presented in the Supplementary Information file. However, The first strip of all the image below was normal mouse liver tissue, which was only used to detect the efficiency of the antibody. Because this study is intended to study the role of HBSP in hepatic ischemia-reperfusion injury, so it focused on comparing the differences between HBSP group and I / R group in liver ischemia-reperfusion injury, and did not focus on HBSP effect on normal mice. So the image we used in the article is that we use the photoshop cut off the first strip of the image.

**Figure2-LC3**

From left to right: normal,I/R, HBSP

**
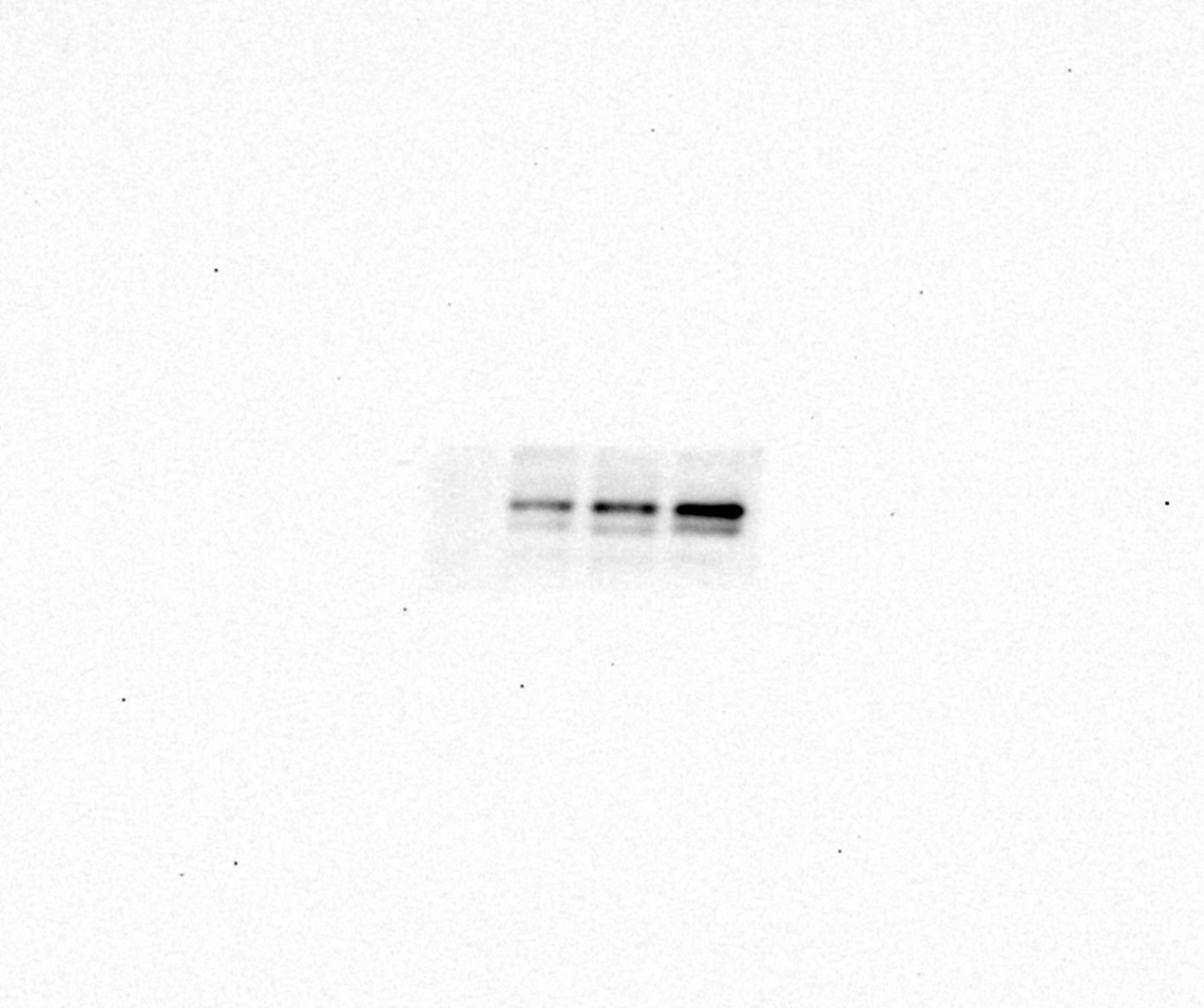
**

**Figure1-Beclin1**

From left to right**:** normal, I/R, HBSP

**
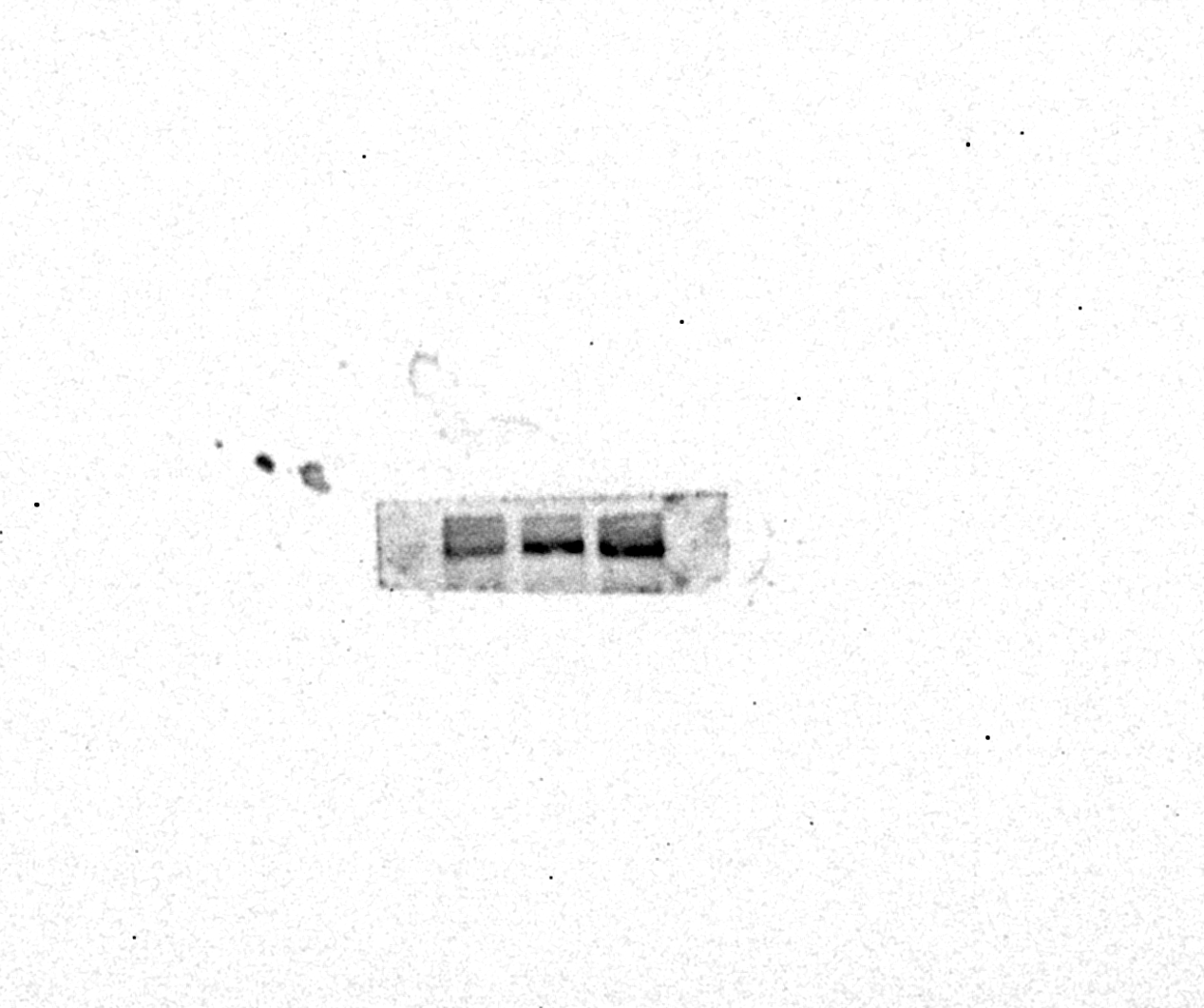
**

**Figure2-beta-actin**

From left to right: normal, I/R, HBSP

**
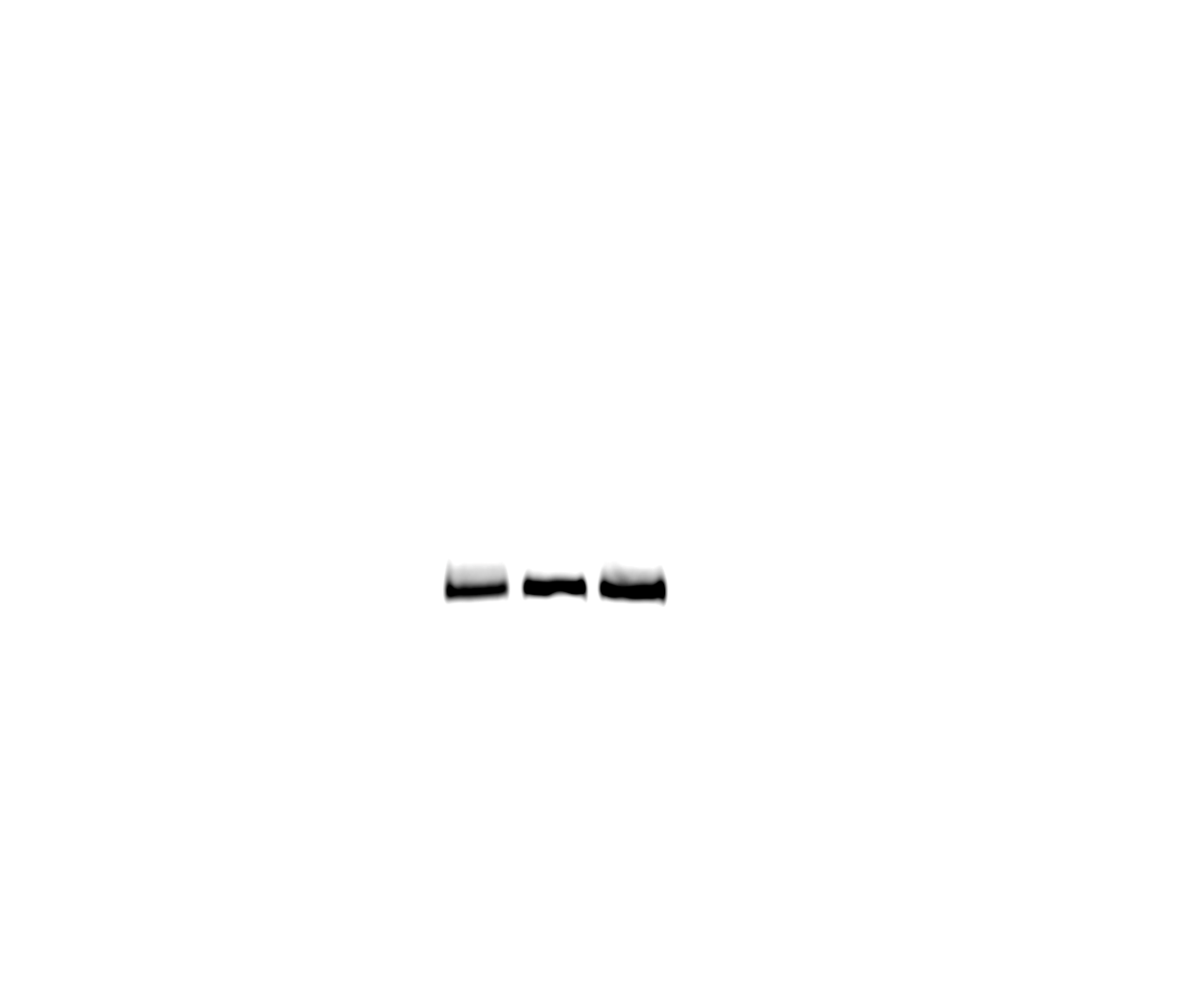
**

**Figure3-LC3**

From left to right: normal,I/R,HBSP,HBSP+3-MA

**
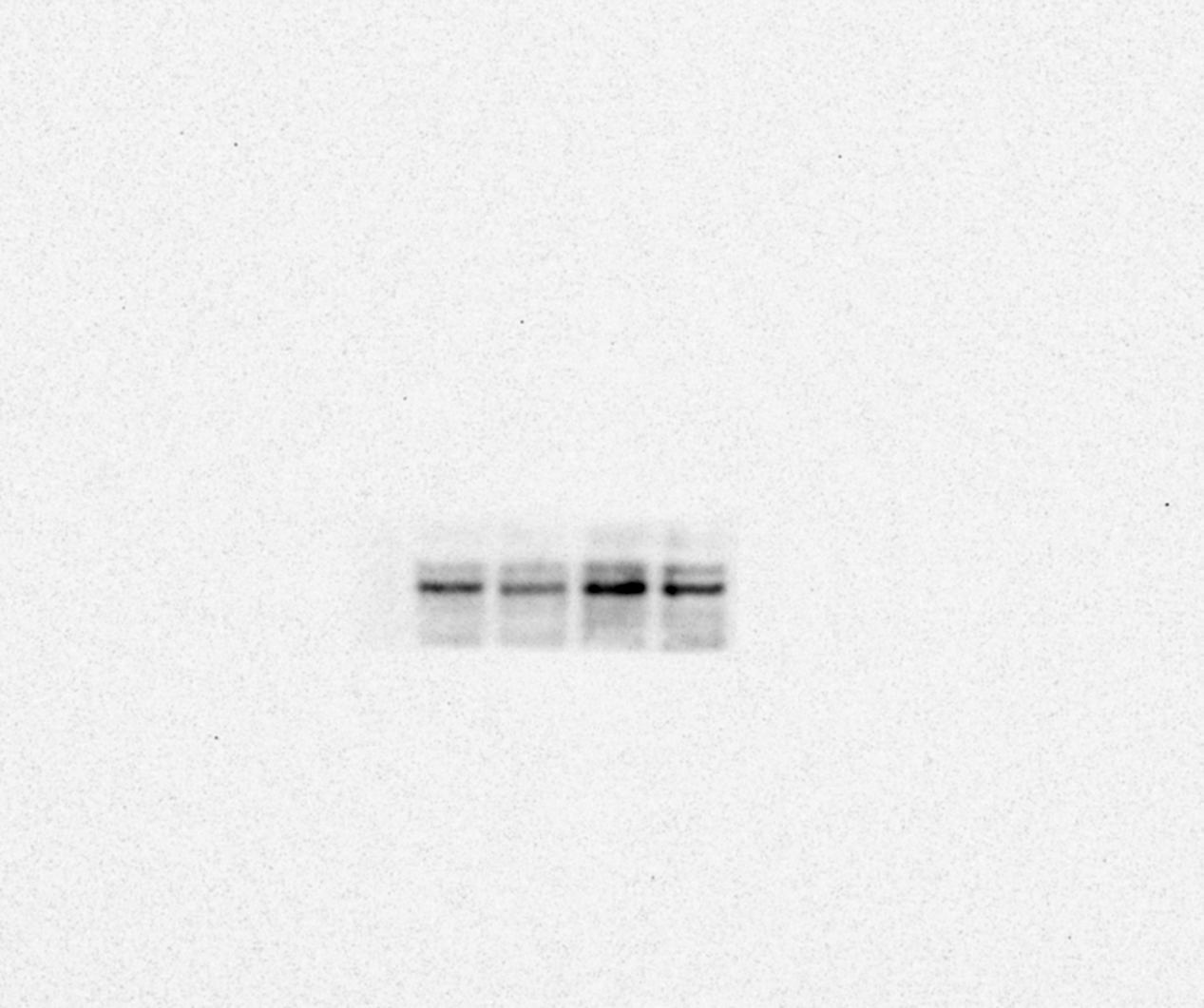
**

**Figure3-Beclin-1**

From left to right:normal, I/R, HBSP, HBSP+3-MA

**
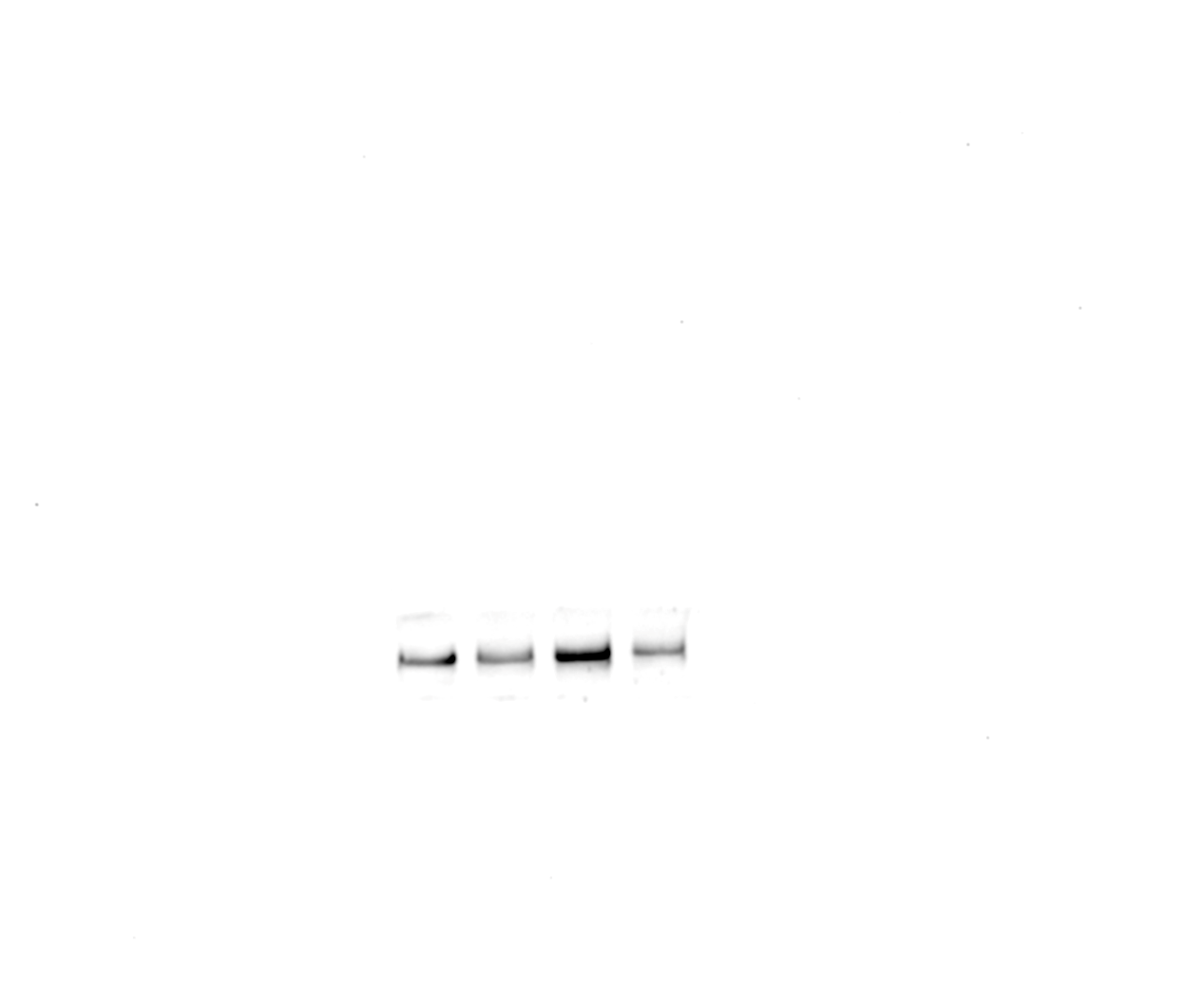
**

**Figure3-beta-actin**

From left to right: normal, I/R, HBSP, HBSP+3-MA

**
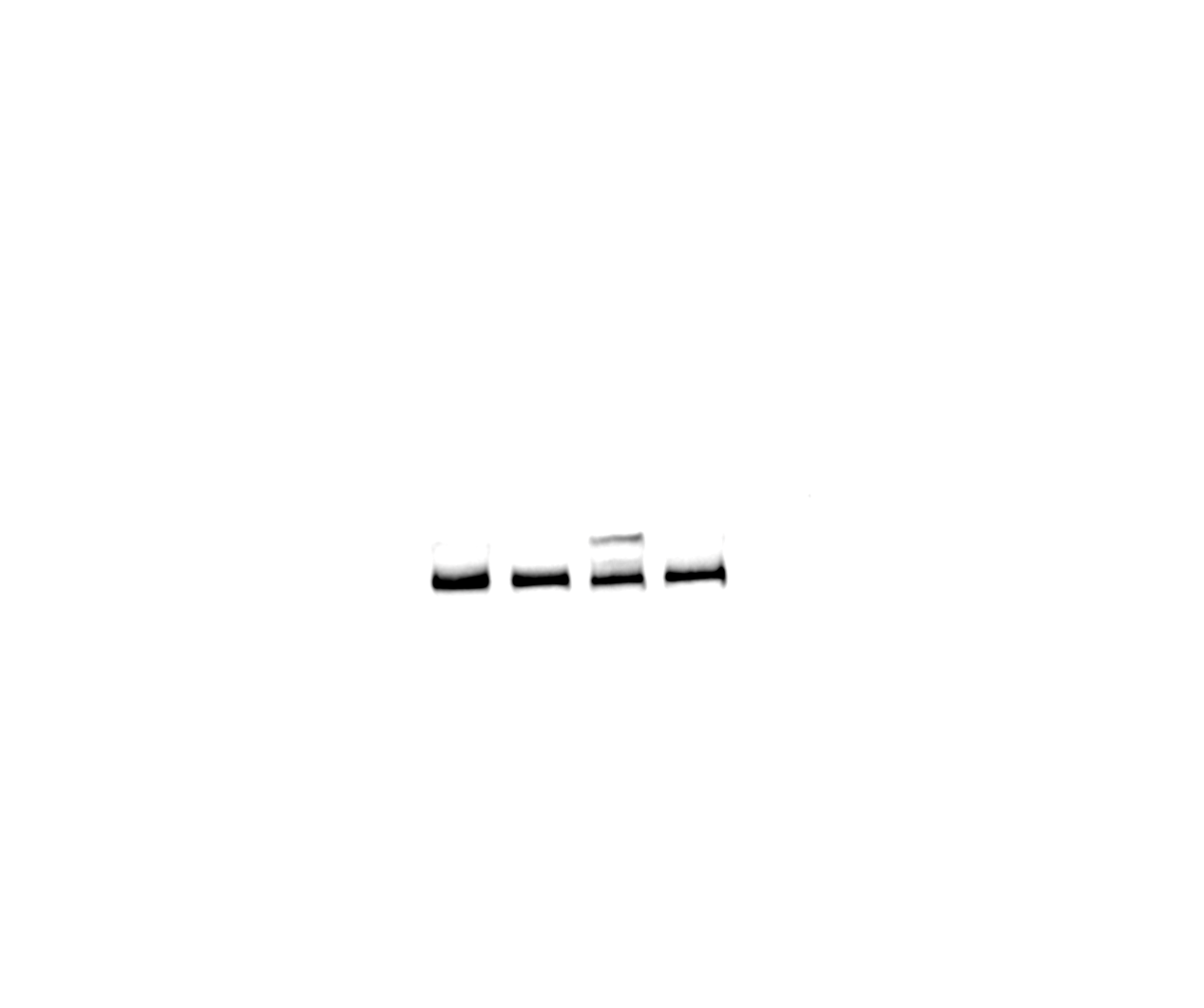
**

**Figure4A-p-mTOR**

From left to right :normal, I/R, HBSP

**
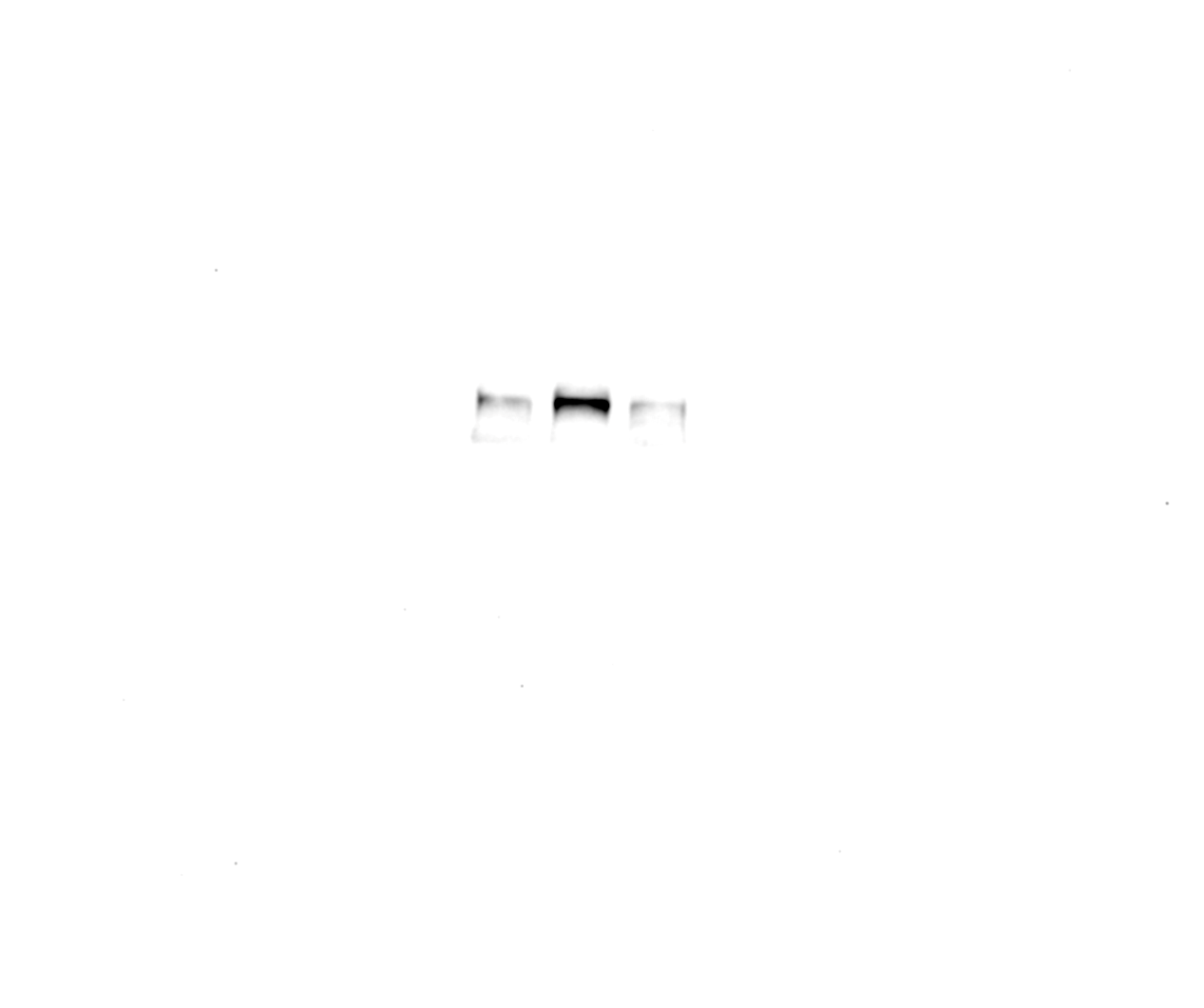
**

**Figure4A-mTOR**

From left to right:normal,I/R, HBSP

**
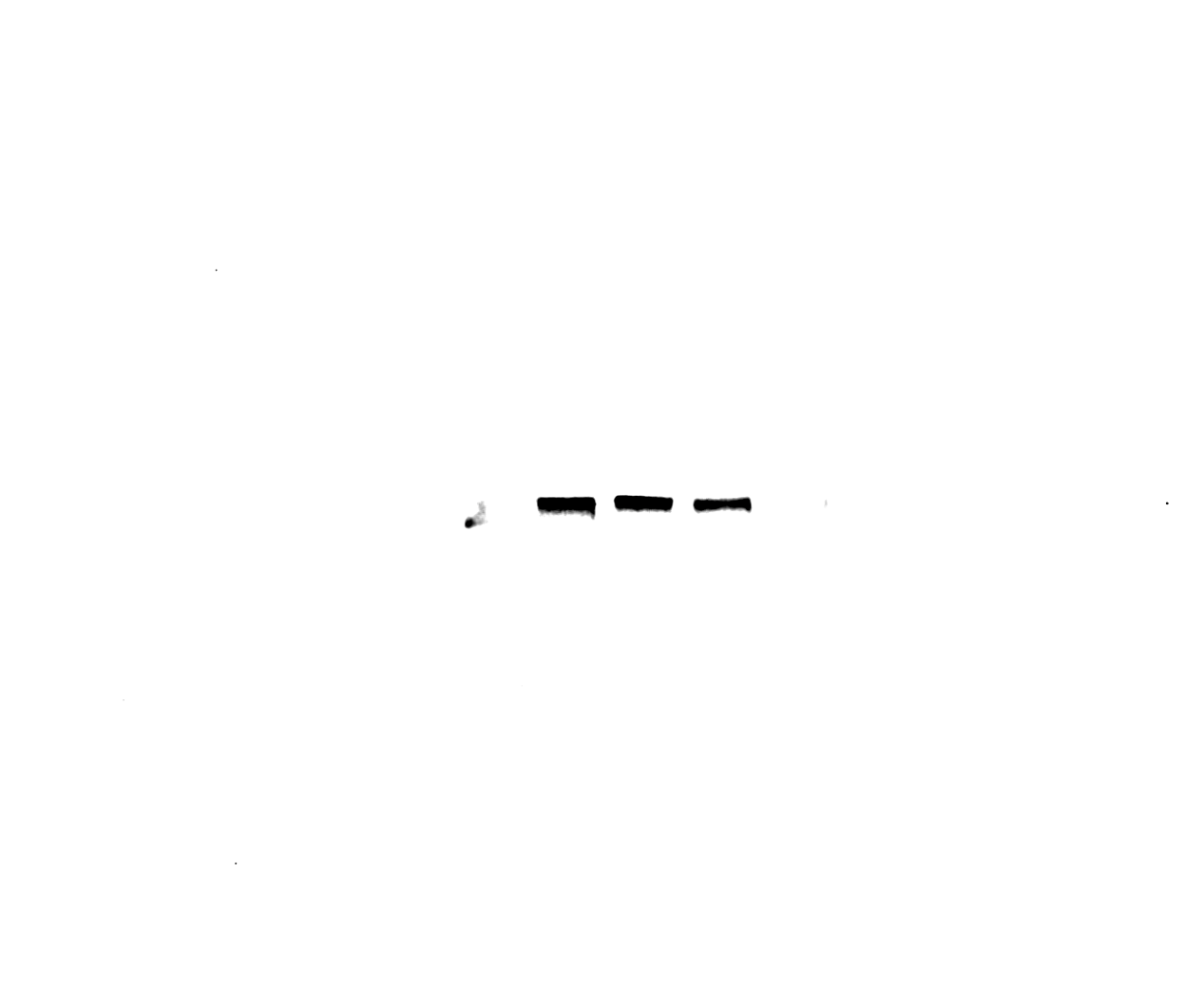
**

**Figure4B-p-mTOR**

From left to right :normal, I/R, HBSP, HBSP+Rapamycin

**
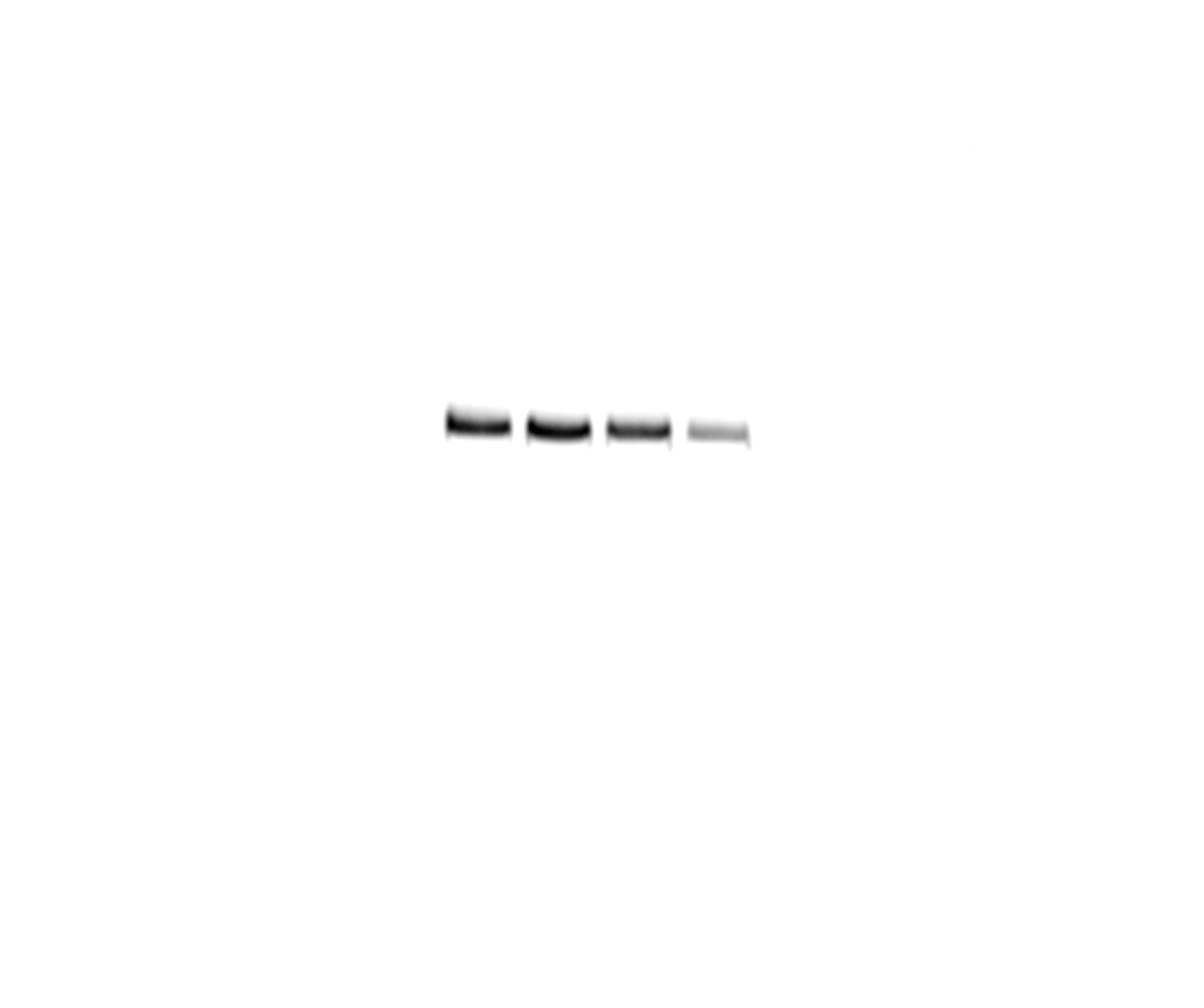
**

**Figure4B-mTOR**

From left to right: normal, I/R, HBSP, HBSP+Rapamycin

**
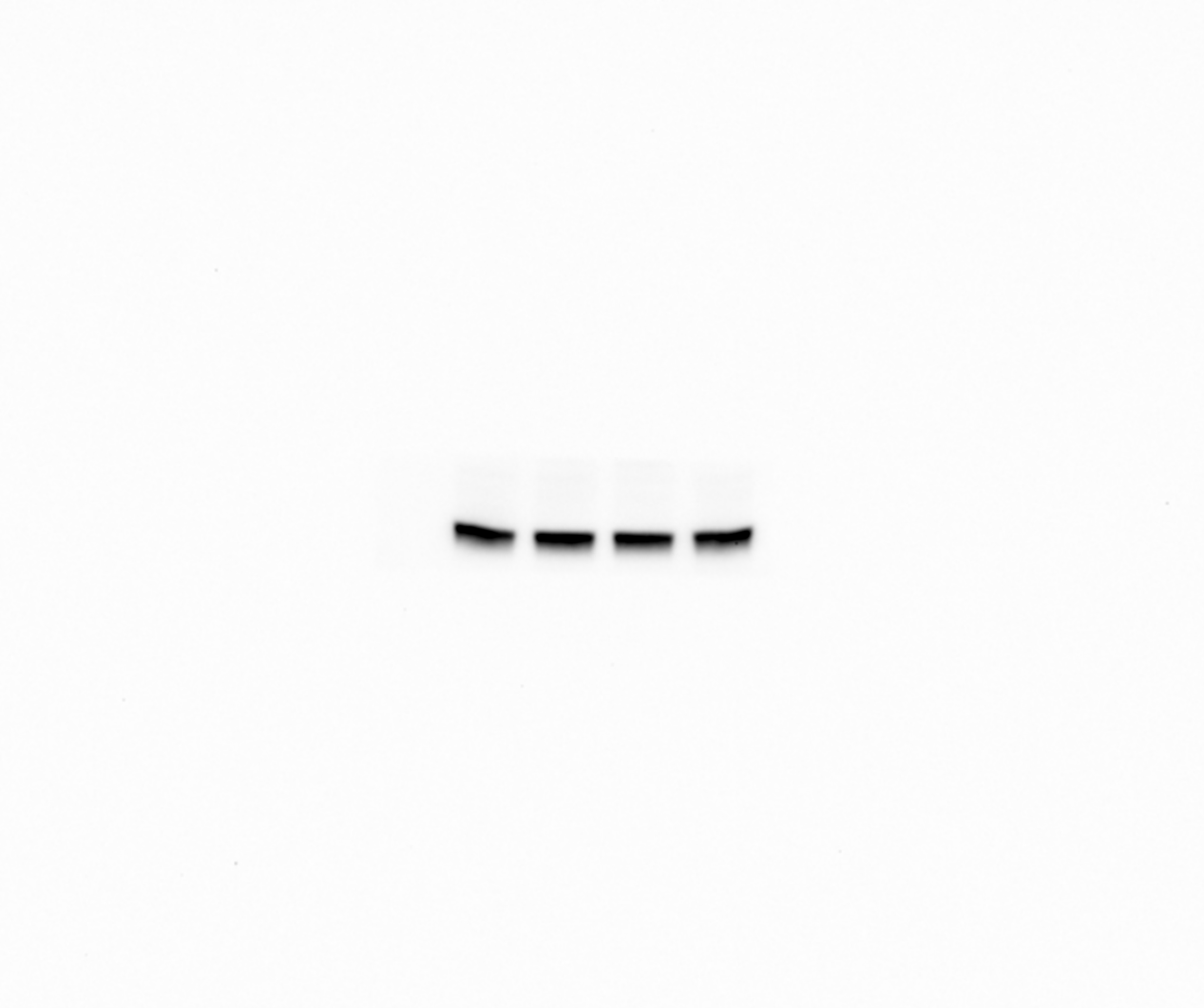
**

**Figure4B-LC3**

From left to right: normal, I/R, HBSP, HBSP+Rapamycin

**
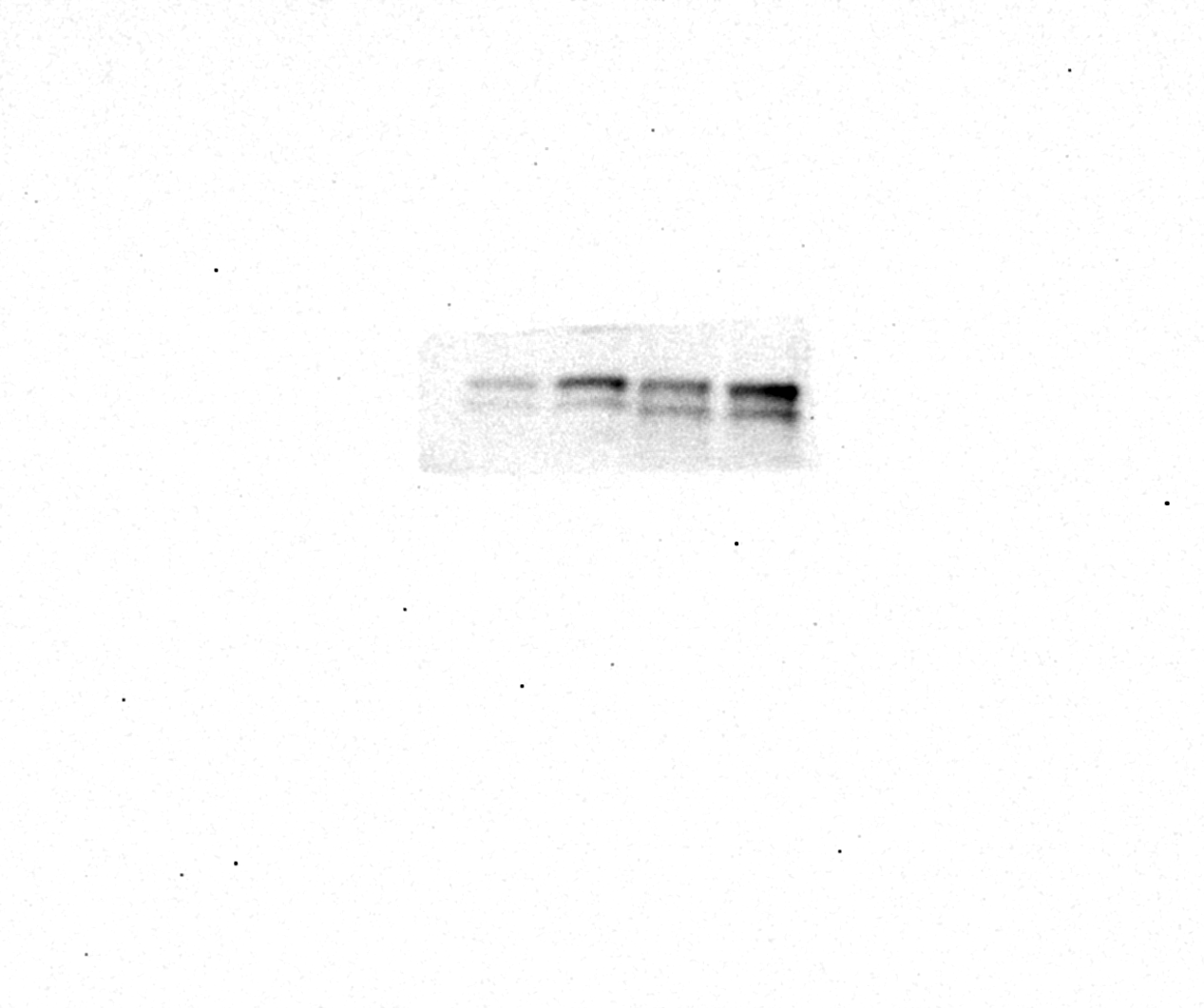
**

**Figure4B-Becline-1**

From left to right: normal, I/R, HBSP, HBSP+Rapamycin

**
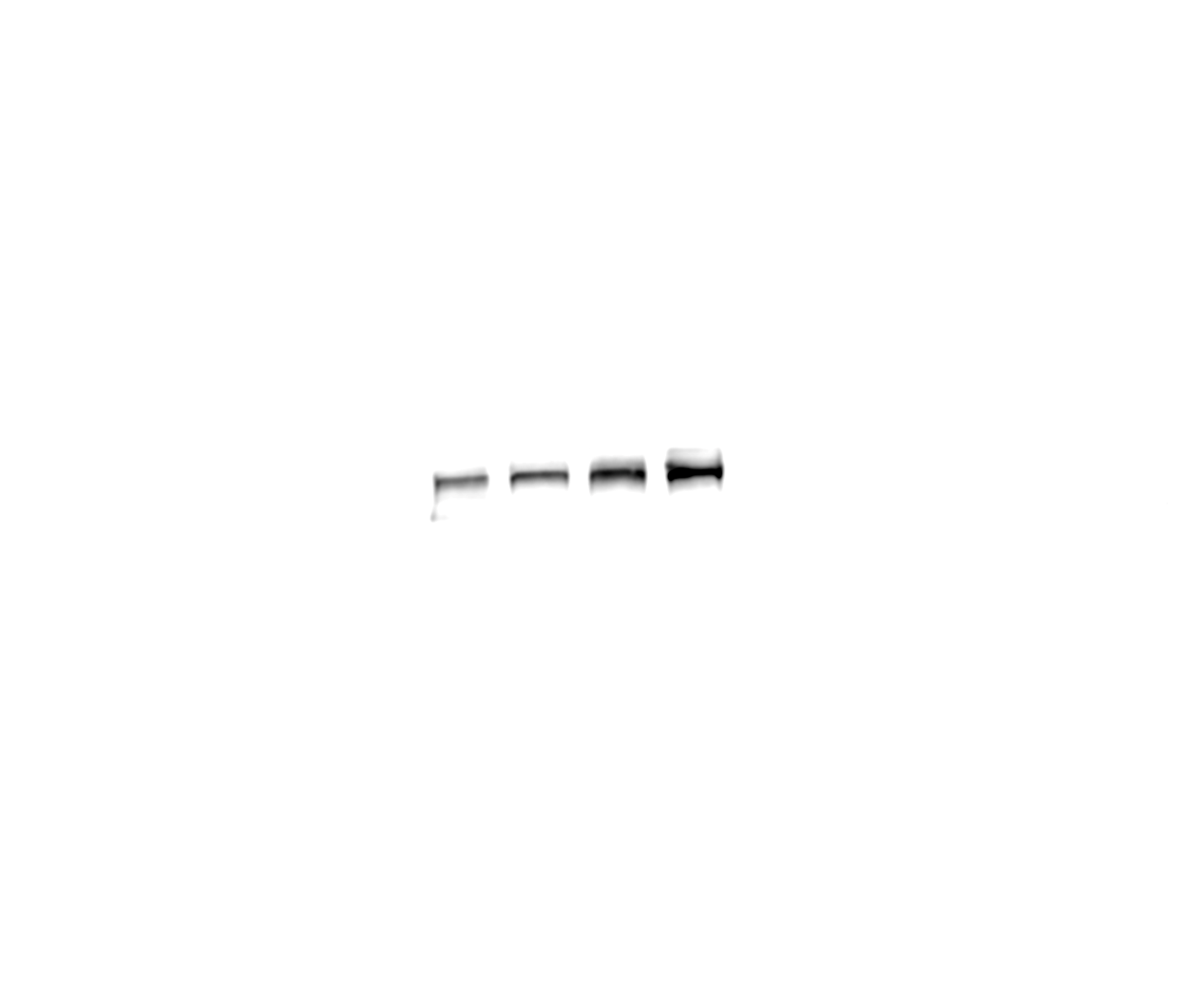
**

**Figure4B-beta-actin**

From left to right: normal, I/R, HBSP, HBSP+Rapamycin

**
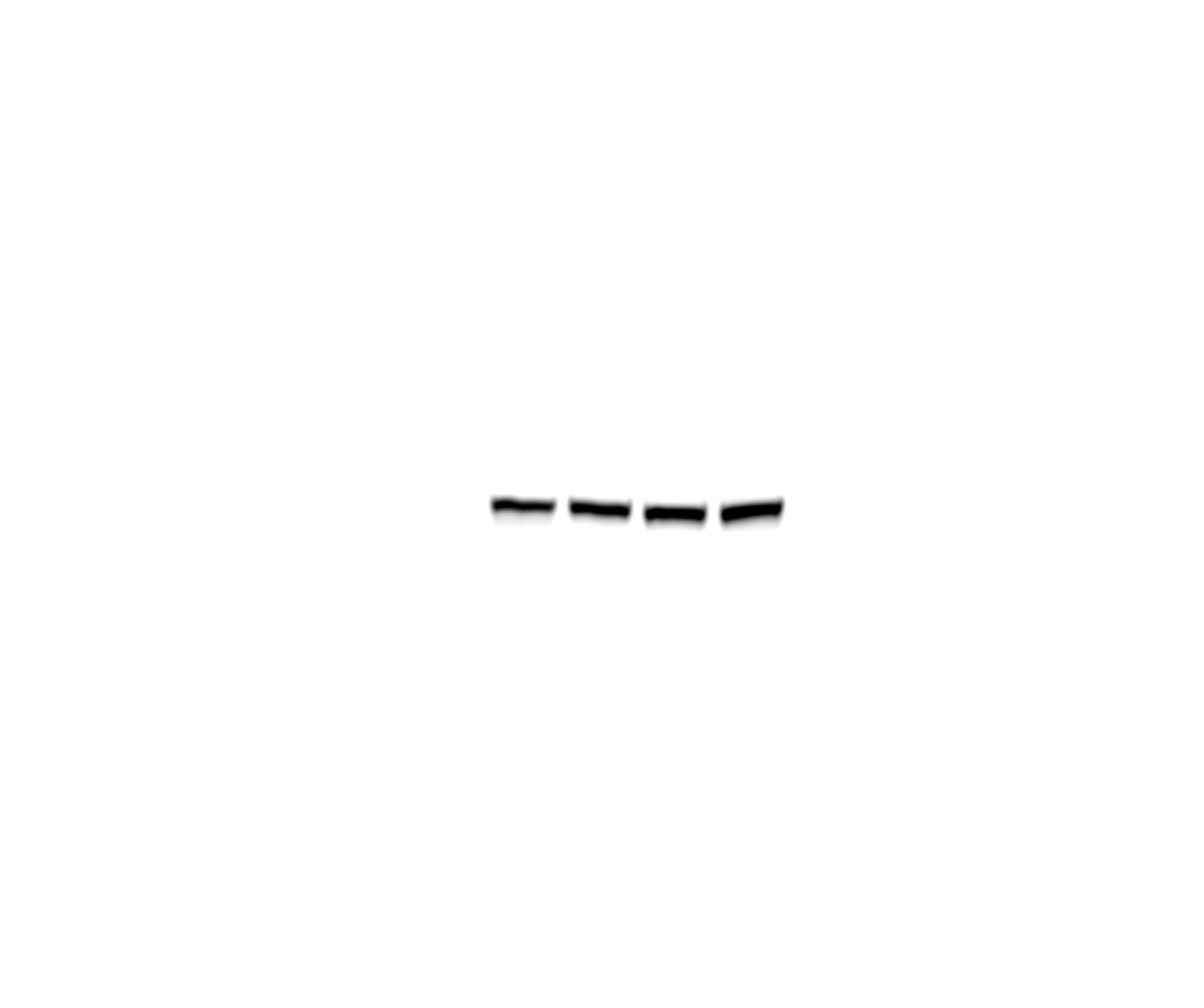
**

**Figure5-p-AKT**

From left to right: normal, I/R, HBSP, HBSP+LY294002

**
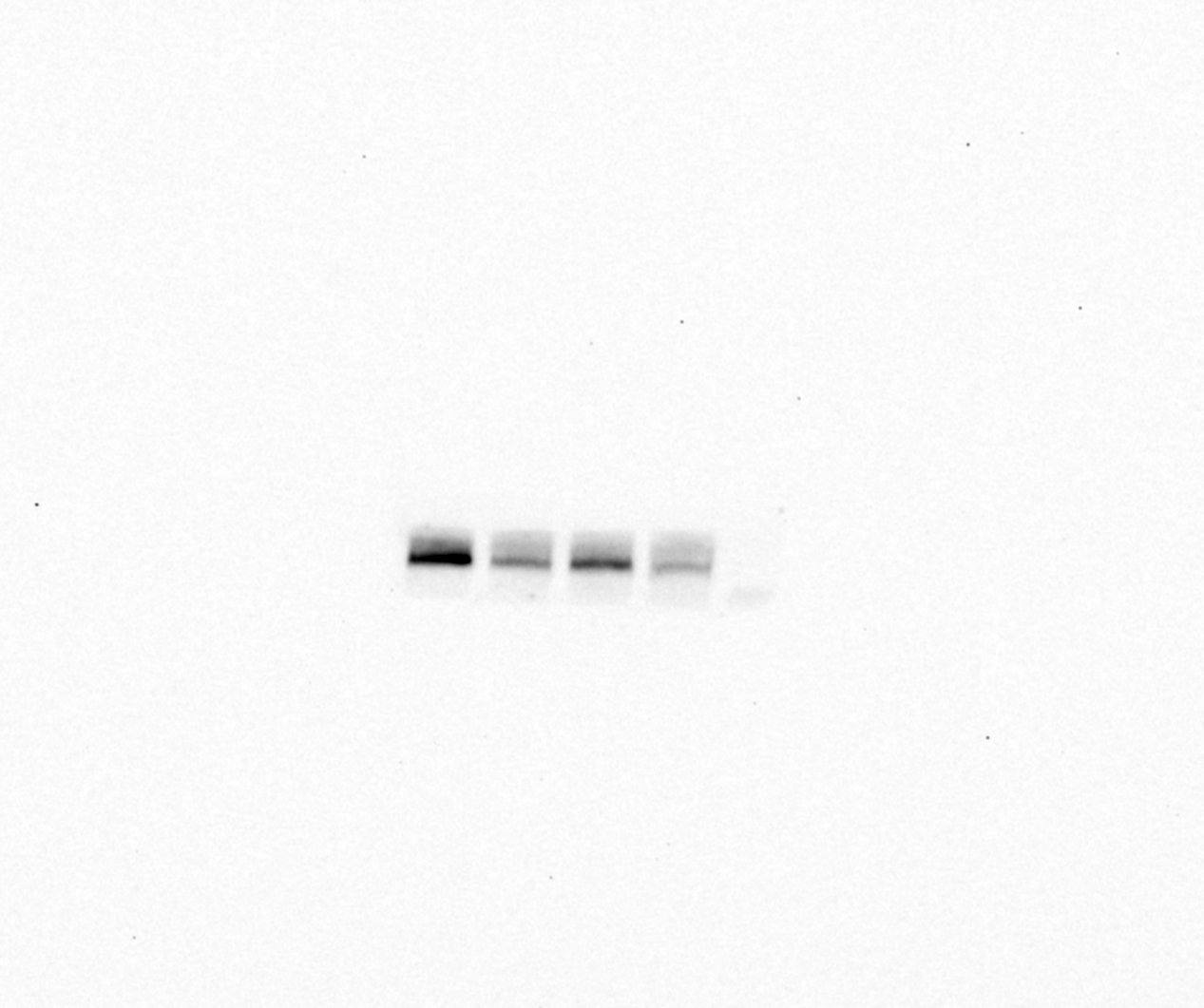
**

**Figure5-AKT**

From left to right: normal, I/R, HBSP, HBSP+LY294002

**
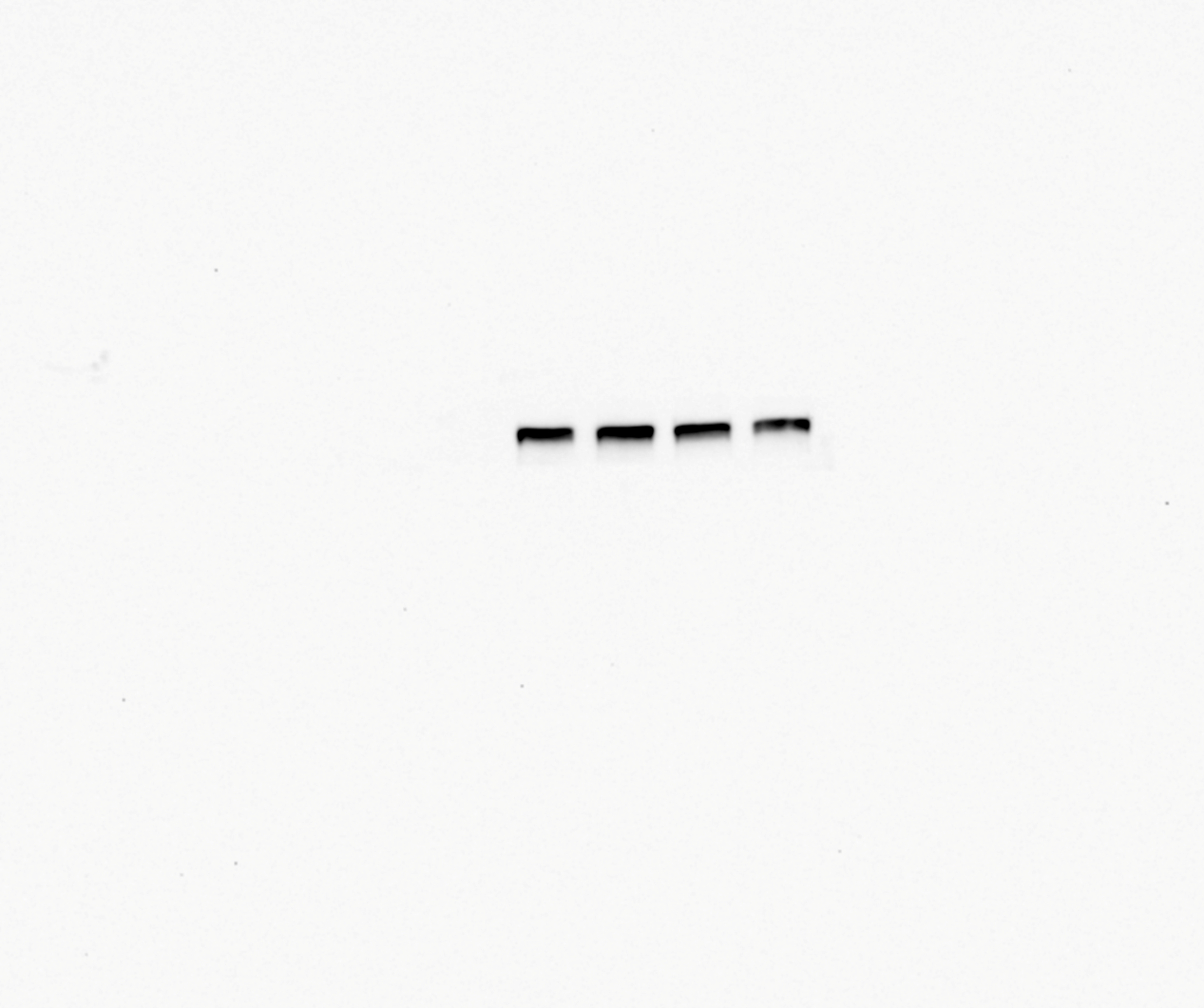
**
